# Comparison of computed tomography and guided bronchoscopy in the diagnosis of pulmonary nodules: A systematic review and meta-analysis

**DOI:** 10.1515/med-2024-1108

**Published:** 2025-07-11

**Authors:** Yilin Wang, Xianhe Liang, Haitao Zhang, Jinhua Hao, Min Wei

**Affiliations:** Department Radiological, Zhongshan Torch Development Zone People’s Hospital, Zhongshan, 528437, China; CT Room, Zhongshan Torch Development Zone People’s Hospital, Zhongshan, 528437, China

**Keywords:** computed tomography, bronchoscopy-guided biopsy, pulmonary nodules, diagnosis, accuracy

## Abstract

**Objective:**

To compare the diagnostic effects of computed tomography (CT) and bronchoscopy guided biopsy on pulmonary nodules.

**Methods:**

“Subject words + free words” were used to search four literature databases including Pubmed, Embase, Cochrane Library, and Web of Science. The subject words were CT, bronchoscope-guided biopsy, pulmonary nodules, and diagnosis. The search is up to August 25, 2023. The retrieved literature was screened, and the literature with duplicate, review, incomplete data or article content and inconsistent with our research was deleted. Revman 5.4 was used for bias analysis of the literatures included in the analysis, and forest map, funnel map, and ROC curve were drawn to compare the accuracy, specificity, and sensitivity of the two diagnostic methods.

**Results:**

A total of 2,622 articles were retrieved, and 8 articles were included in the study after screening. Bias analysis and funnel plot showed that eight included articles had higher quality and smaller bias. The sensitivity and specificity of the forest map were both 95% CI, and the sensitivity of CT diagnosis was better than that of bronchoscopy diagnosis, with similar specificity between the two groups. The funnel plot shows some heterogeneity in the literature on pulmonary nodules. The ROC curve shows that CT diagnosis is significantly superior to bronchoscopy diagnosis, with diagnostic accuracy approaching 100%.

**Conclusion:**

CT has significant advantages in detecting and diagnosing pulmonary nodules, as it can detect nodules in various parts of the lungs. However, bronchoscopy has significant advantages in diagnosing small pulmonary nodules.

## 1 Introduction

Computed tomography (CT) is the use of precisely collimated X-ray beam, gamma ray, ultrasonic wave, and zero-density detector together around a certain part of the human body for one by one tomography scan, with the characteristics of fast scanning and clear images [1,2]. With the development of CT technology and the wide application of high-resolution CT in physical examination, more and more patients have pulmonary nodules, and the differential diagnosis of pulmonary nodules is becoming more and more important [3,4]. Pulmonary nodules are well-delimit, round or irregular shapes with a diameter of less than 30 mm that can be seen on chest CT [5]. Pulmonary nodules are divided into small nodules (diameter < 5 mm), small nodules (5 mm ≤ diameter < 10 mm), and nodules (diameter ≥ 10 mm) [6,7]. According to the density of nodules, they can be divided into solid nodules, ground glass nodules, and partial solid nodules [8]. The cause of pulmonary nodules is likely to be irritation by air environmental factors, occupational exposure, lung infection, abnormal inflammatory response, tumor, etc. [4] The pathological basis is thickening of the alveolar septum or partial filling of the alveolar cavity with fluid, cells, or tissue debris [9]. The appearance of pulmonary nodules suggests that the body may suffer from inflammation, benign tumors, malignant tumors, pulmonary interstitial diseases, pulmonary lymph nodes, etc. Generally, pulmonary nodules are inflammatory nodules, granulomas, pneumoconiosis nodules, inflammatory pseudotumors, tuberculous spheres, vascular lesions, or malignant tumors, and most of them are benign nodules [10,11]. Clinically, nodules <5 mm are low-risk nodules and patients need regular follow-up, while nodules <3 mm do not need follow-up [12]. Nodules >8 mm are high-risk and require 3 months follow-up or needle biopsy and pathological diagnosis [13].

Bronchoscopy is a long and thin bronchoscope inserted into the lower respiratory tract of the patient after local anesthesia through the mouth or nasal cavity, and the lesions of the patient’s trachea and bronchus can be directly observed [14]. The commonly used bronchoscopes are rigid bronchoscopy, soft bronchoscopy, flexible bronchoscopy, and fiber bronchoscopy. Bronchoscopy cannot diagnose the nature of pulmonary nodules, so it is necessary to cooperate with biopsy, i.e., to manually remove a little lung tissue under bronchoscopy for pathological examination, in order to confirm the diagnosis [15]. In this study, we conducted a systematic review and meta-analysis of the diagnosis of pulmonary nodules by CT and bronchoscopy, comparing their diagnostic accuracy and sensitivity.

## 2 Methods

### 2.1 Study design

We conducted this study between August 24, 2023 and September 10, 2023. This study is a meta-analysis of comparison of CT and guided bronchoscopy in the diagnosis of pulmonary nodules. First, relevant literatures were retrieved from four databases, and then the literatures included in the analysis were selected. Finally, meta-analysis software Review Manager 5.4 was used to analyze the included literatures, and a good imaging method (CT) for the diagnosis of pulmonary nodules was obtained.

### 2.2 Literature search and screening

Literature search was conducted on Pubmed, Embase, Cochrane Library, and Web of Science. The deadline is August 26, 2023. Search in the form of subject word + free word, and the search content is “(((pulmonary nodules) OR (Lung Neoplasms) OR (Multiple Pulmonary Nodules) OR (Solitary Pulmonary Nodule)) AND ((computed tomography) OR (Single Photon Emission Computed Tomography Computed Tomography) OR (Multidetector Computed Tomography)) OR (Four-Dimensional Computed Tomography) OR (Spiral Cone-Beam Computed Tomography)) OR (Cone-Beam Computed Tomography) OR ((Positron Emission Tomography Computed Tomography)) AND (guided bronchoscopy) AND (diagnosis)))”.

All literatures were screened independently by more than two researchers. In case of disagreement, a third researcher was invited to make a review. The inclusion criteria were (1) publicly published literature, (2) no language restriction, (3) the full text of the literature can be obtained and the data are complete, and (4) literature on the diagnosis of pulmonary nodules by CT or bronchoscopy. Exclusion criteria were (1) the duplicate literature, (2) the review, meta-analysis, (3) literature that could not obtain the full text, (4) literature that was not related to the diagnosis of pulmonary nodules by CT or bronchoscopy, and (5) the literature with incomplete data.

### 2.3 Basic information of the included literature

The information of the included literature was extracted, including: author, publication year, language, country, number of subjects, gender, age, detection method, true positive (TP), false positive (FP), true negative (TN), false negative (FN), and pathological diagnosis results. The table to list the above information was made.

### 2.4 Bias situation

Bias analysis was performed on the included literature in the Review Manager 5.4 (Revman 5.4) software, each included literature was evaluated in the following six aspects: random sequence generation (selection bias), allocation consideration (selection bias), blinding of participants and personnel (performance bias), blinding of output assessment (detection bias), incomplete output data (attrition bias), selective reporting (reporting bias), and other bias. The bias analysis graph was ultimately generated by Revman 5.4 software.

### 2.5 Forest map

Revman 5.4 software was used to analyze the true positive, false positive, true negative, and false negative data of CT and bronchoscopy diagnosis in all the included literatures, and forest maps were made to describe the sensitivity and specificity of diagnosis, in which the specificity reflected the diagnostic accuracy. That is, a line segment with endpoints 0 and 1 is taken as the horizontal axis, and multiple line segments parallel to the horizontal axis are used to describe the effect size and confidence interval (CI) of each included study, and a prism is used to describe the effect size and CI of multiple studies combined.

### 2.6 Funnel plot

The funnel diagrams were made with Revman 5.4 software. The funnel plot is a scatter plot that visually reflect the estimated intervention effects of individual studies with a given sample size or accuracy, and can be used to identify publication bias and other biases. In the results of funnel plot, the larger the sample size, the smaller the error and the smaller the bias, the higher the scatter distribution; if not, go lower.

### 2.7 Receiver operating characteristic (ROC) curve

Each point on the ROC curve reflects the sensitivity to the same signal stimulus. ROC curve used false positive rate (FPR) specificity as the horizontal axis to classify the proportion of all negative examples in a specific instance (1-specificity), and true positive rate (TPR) sensitivity as the vertical axis. That is, positive coverage (sensitivity). There are four diagnostic results for pulmonary nodules: TP, FP, TN, and FN. The higher the TPR and the lower the FPR, the better the prediction ability of the model.

### 2.8 Data statistics

Determine accuracy and specificity were based on the results of CT and bronchoscopy examinations, as well as patient biopsy or postoperative pathological diagnosis. The true positive, false positive, true negative, and false negative diagnoses in each included literature were collected and calculated. The closer the 95% CI values for sensitivity and specificity in the forest map are to 1, the better. The larger the aspect under the ROC curve, the better.

## 3 Results

### 3.1 Literature screening

**Figure 1:**
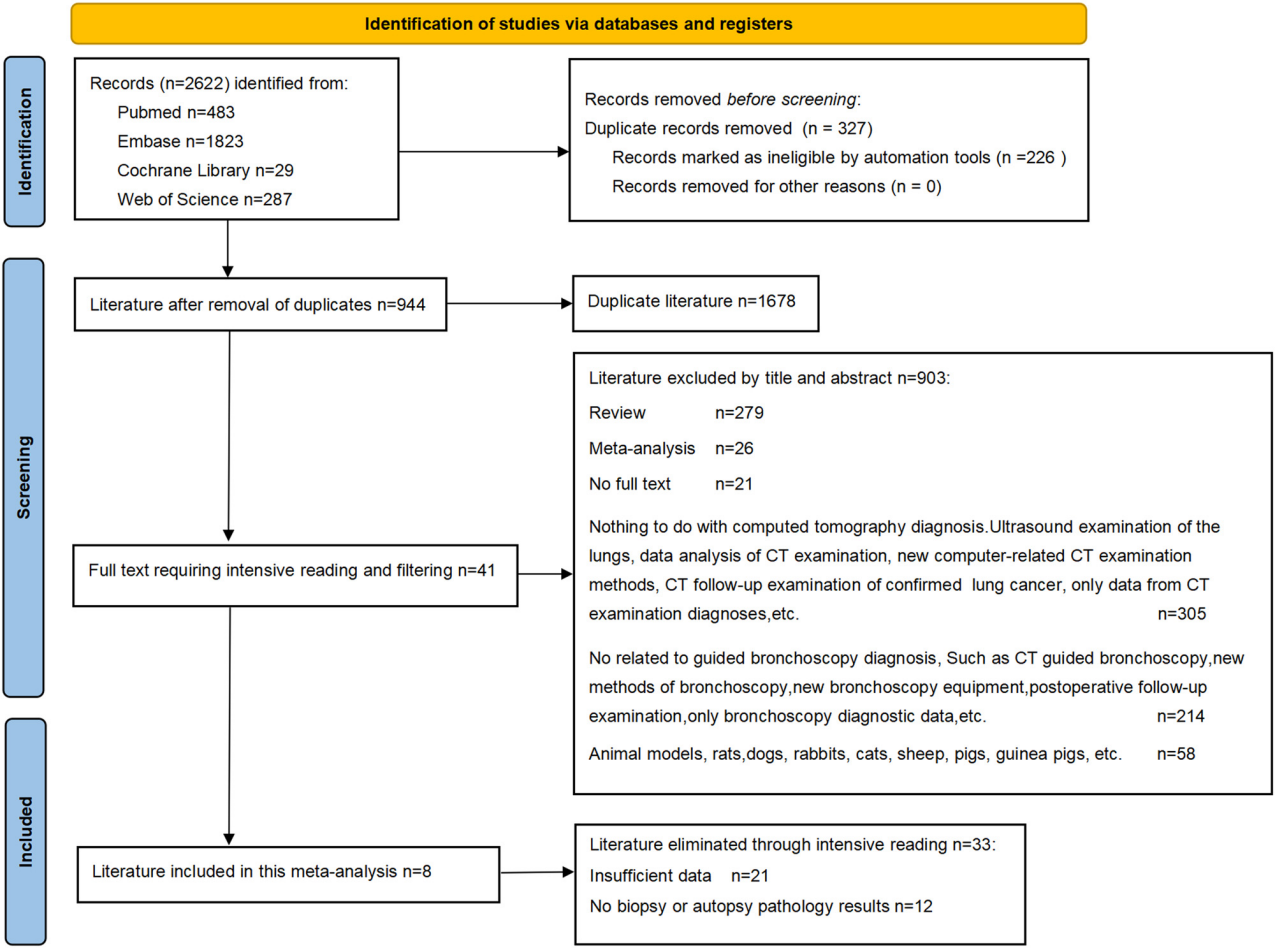
Literature screening chart. All retrieved literature has undergone three rigorous screenings.

A total of 2,622 articles were obtained by searching four major databases, including 483 in Pubmed, 1,823 in Embase, 29 in Cochrane Library, and 287 in Web of science. There were 1,678 duplicate literatures deleted, such as 279 reviews, 26 meta-analyses, 21 incomplete literatures, 305 literatures unrelated to CT diagnosis or only bronchoscopy results, 214 literatures unrelated to bronchoscopy or only bronchoscopy results, 58 animal-related literatures, and 21 literatures with incomplete data. There were 8 literatures without biopsy or autopsy pathological results, and 12 literatures were finally included. These 12 articles all describe the comparison of CT and bronchoscopy in the diagnosis of pulmonary nodules (Figure 1). The results of CT diagnosis and bronchoscopy diagnosis from the included authors, countries, languages, and literature studies were tabulated for comparison, as shown in Table 1.

**Table 1:** Basic information of included literature and included research

Author and year	Country	Research type	Diagnostic time	Diagnostic institution	Diagnostic method (Group 1 vs Group 2)	Total number of patients	Number of patients (Group 1 vs Group 2)	Age, years (Group 1 vs Group 2)	Sex (Group 1 male/female vs Group 2 male/female)	BMI, kg/m^2^ (Group 1 vs Group 2)	Nodule size range	Positive diagnostic rate (Group 1 vs Group 2)	Sensitivity (Group 1 vs Group 2)	Specificity (Group 1 vs Group 2)	Whether the positive rate of diagnosis was statistically significant	Complications (Group 1 vs Group 2)
Zhang S 2023	China	Retrospectively evaluated	Between June 2021 and June 2022	Fujian Medical University Union Hospital	Electromagnetic navigation bronchoscopy (ENB) technique vs computed tomography (CT)-guided lung puncture	114	21 vs 93	(60.76 ± 10.67) vs (62.18 ± 11.33)	12(57.1%)/9(42.9%) vs 49 (52.6%)/44(47.4%)	(22.68 ± 2.42) vs (23.23 ± 3.67)	Maximum nodule diameter of ≥8 mm	76.1 vs 73.6%	Not mention	Not mention	No statistical significance	None vs pneumothorax incidence, tube placement, postoperative hemorrhage, and symptomatic hemorrhage rates were 16.1, 6.5, 6.5, and 1.1%
Yu Lee-Mateus A 2023	United States	A multicenter retrospective study	Between January 2019 and March 2021	Mayo Clinic Florida and Mayo Clinic Rochester, United States	Robotic-assisted bronchoscopy (RAB) vs computed tomography-guided transthoracic biopsy (CTTB)	225	113 vs 112	(70 ± 9,8) vs (67.4 ± 12.4)	57(50.4%)/56(49.6%) vs 57(50.9%)/55(49.1%)	(27.9 ± 6.5) vs (27.0 ± 5.2)	(1.0–2.7 cm) vs (0.8–2.6 cm)	87.6 vs 88.4%	82.1 vs 88.5%	100 vs 100%	Not mention	Complication rate was significantly higher for CTTB compared to RAB (17 vs 4.4%; *p* = 0.002)
Styrvoky K 2022	United States	A single-center, retrospective observational study	Between December 2020 and February 2022	UT Southwestern Medical Center in Dallas, Texas	ssRAB with R-EBUS and/or CBCT vs ssRAB only	198 (209 nodules)	114 lesions vs 9 lesions	67.1 ± 12.9	Male, female: 47.5%, 52.5%	Not mention	22.6 ± 13.3 mm (7–73 mm)	91.4%	87.3%	98,7%	Have statistical significance	A low rate of complications
Atkins NK 2020	United States	A retrospective study	From January 2017 to August 2019	A large, academic, tertiary care hospital	Non-diagnostic ENB biopsy vs CT-guided biopsy (CTB) (successively)	135	135 vs 135	62.4 ± 12.8	Male, female: 51%, 49%	Not mention	28.2 ± 16.7 mm	16.7–86.0% vs 100%	Not mention	Not mention	Have statistical significance	Not mention
Wang W 2018	China	A randomized pragmatic trial	June 2014 to June 2016	Nanjing Chest Hospital	R-EBUS) vs CT-guided needle biopsy (CT-PNB)	215	80 vs 80	(58.67 ± 13.55) vs (59.03 ± 13.06)	48(60%)/32(40%) vs 45(56.25%)/35(43.75%)	Not mention	(2.17 ± 0.31) cm vs (2.09 ± 0.30) cm	65 vs 85%	73.7 vs 87.9%	Not mention	Have statistical significance	CT-PNB group were higher than R-EBUS group (*p* = 0.002)
van’t Westeinde SC 2012	Netherlands	Not mention	Between April 2004 and December 2008	Not mention	Conventional bronchoscopy vs CT scan	308	(308j ± 74.2) vs (107 ± 25.8)	62(50–75) vs 62(51–74)	248(80.5%)/60(19.5%) vs 99(92.5%)/8(7.5%)	Not mention	The mean SD diameter of the nodules was 14.6 ± 8.7 mm	7.3 vs 58.6%	13.5 vs 100%	100 vs 100%	Have statistical significance	0.6% (two of 308) of participants and no major complications.
Aristizabal JF 1998	United States	Retrospective review	Between January 1992 and December 1994	At the University of Alabama Hospital and Birmingham Veterans Affairs Medical Center	Fiberoptic bronchoscopy vs CT	64	22 vs 42	64 (38–89)	60(93.75%)/4(6.25%)	Not mention	1.5–10.0 cm	34 vs 75%	Not mention	Not mention	Not mention	Not mention
Shankar S 1998	India	Not mention	Not mention	Not mention	Transbronchial fine needle aspiration (TBNA) vs CT- or fluoroscopy-guided percutaneous transthoracic needle aspiration	30	16 vs 18	2–80	20(66.67%)/10(33.33%)	Not mention	<6 cm	75 vs 78%	Not mention	Not mention	Have statistical significance	No complications vs two patients (11%) had minimal asymptomatic pneumothorax

### 3.2 Bias

**Figure 2:**
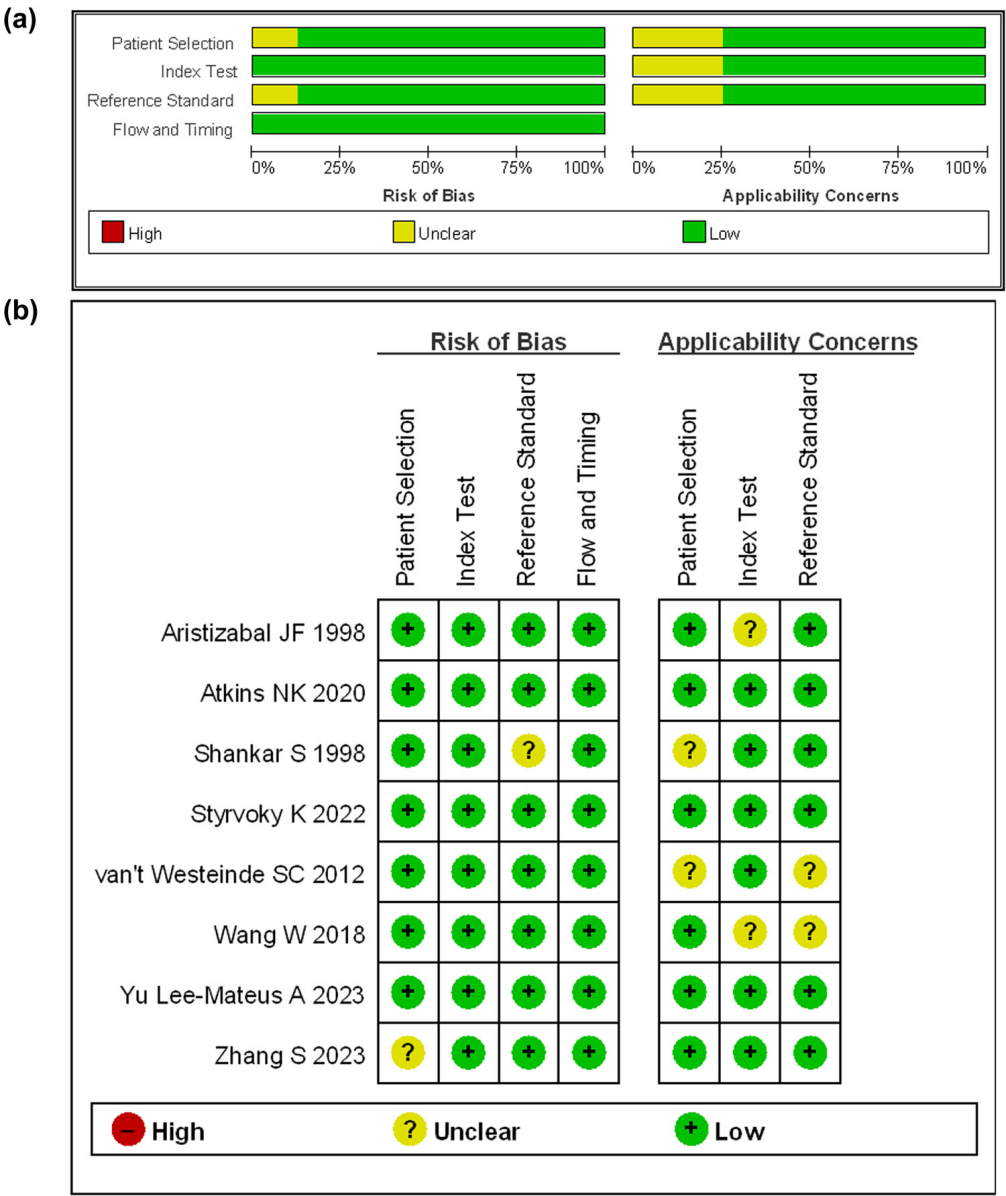
Bias plot: (a) the overall bias of the eight included literature and (b) includes information on each bias issue in the literature.

A bias analysis on four aspects of the eight included literature were conducted, namely patient selection, index test, reference standard, and flow and timing. Green and (−) indicate low bias risk, red and (+) indicate high bias risk, and yellow and (?) indicate unclear. The results are shown in Figure 2a and b.

### 3.3 Forest plot

**Figure 3:**
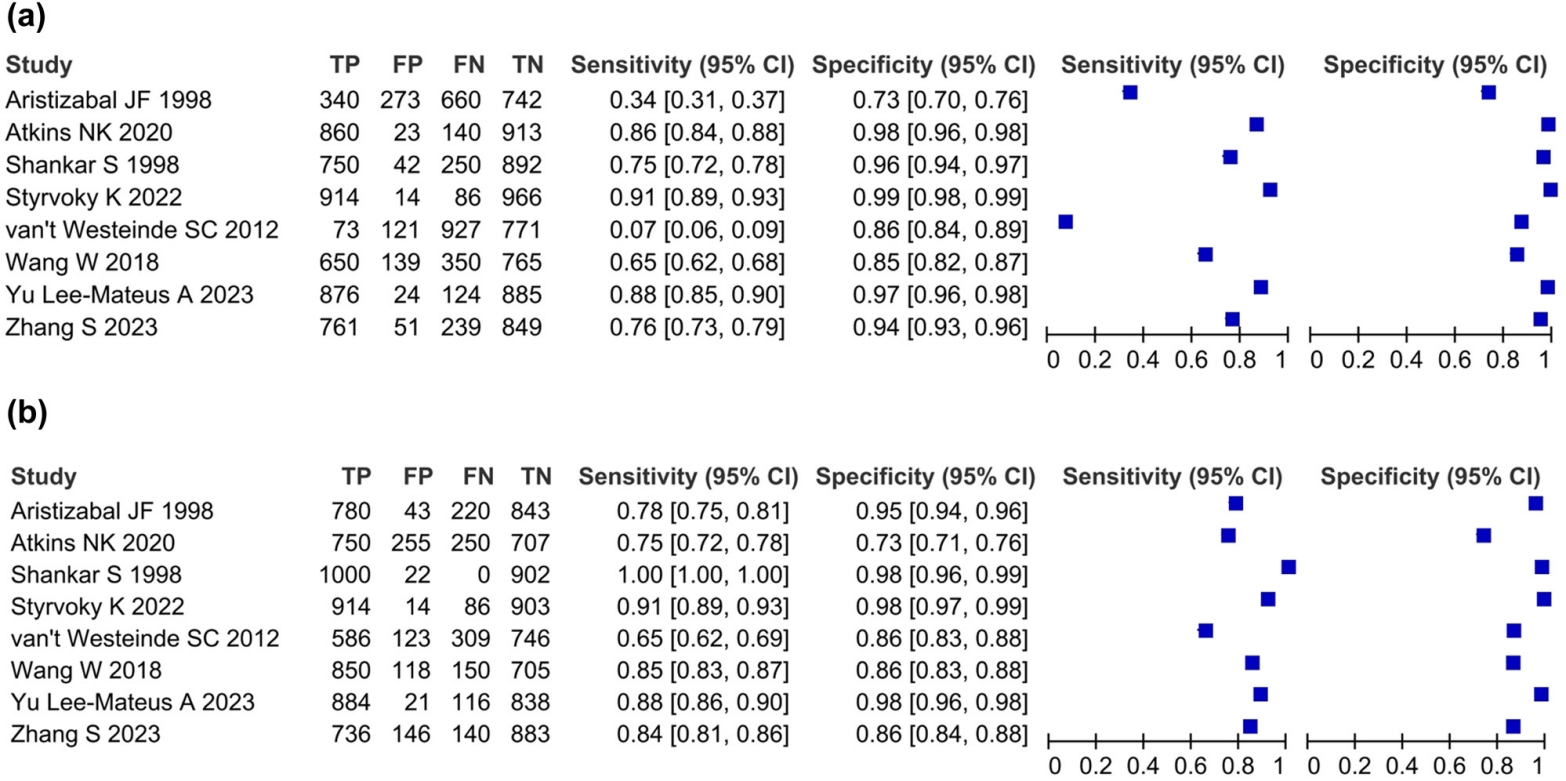
Forest plot: (a) the diagnostic sensitivity and heterogeneity of bronchoscopy for pulmonary nodules in eight included literature, And (b) the diagnostic sensitivity and heterogeneity of CT for pulmonary nodules in eight included literature.

The sensitivity of bronchoscopy diagnosis is significantly lower than that of CT diagnosis (95% CI), and the heterogeneity is not significantly different from CT diagnosis. The sensitivity of the two groups in most studies is close to 1, and the heterogeneity is also close to 1 (Figure 3).

### 3.4 Funnel plot

**Figure 4:**
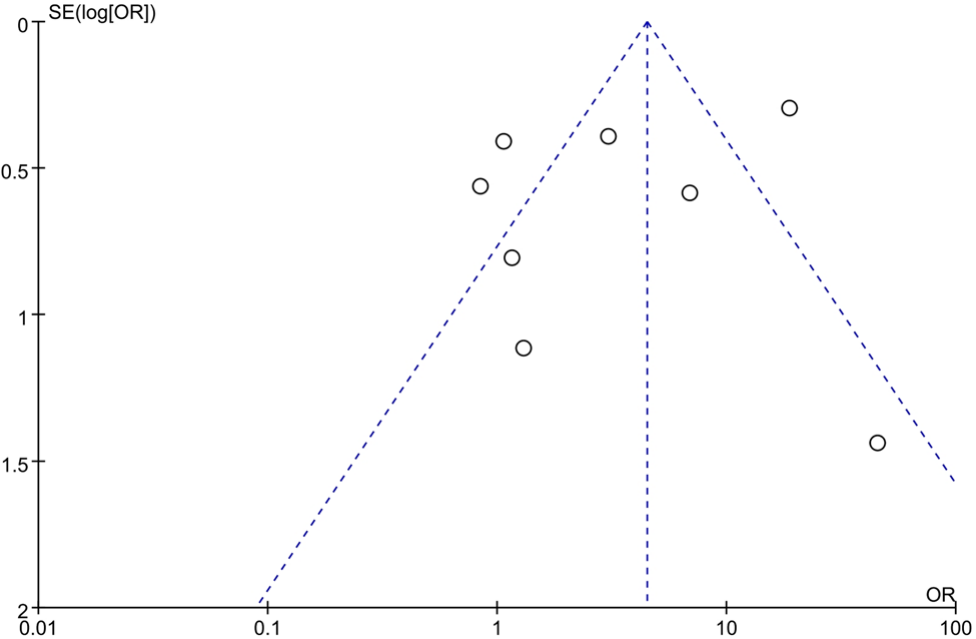
Funnel plot.

The funnel plots of eight studies are basically clustered in the middle, and studies involving small pulmonary nodules or only diagnosing large nodules >3 cm are distributed outside the funnel plots, indicating a certain degree of heterogeneity in the studies (Figure 4).

### 3.5 ROC curve

**Figure 5:**
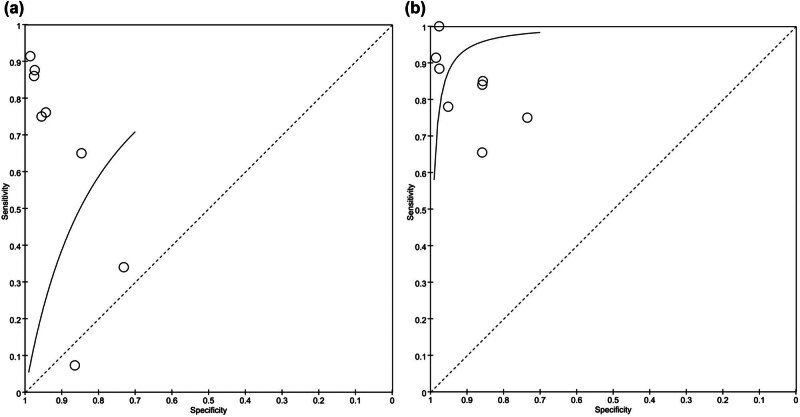
ROC curve: (a) the diagnostic accuracy of bronchoscopy for pulmonary nodules and (b) the diagnostic accuracy of CT for pulmonary nodules.

The diagnostic accuracy of bronchoscopy under the ROC curve is significantly lower than that of CT diagnosis. As shown in Figure 5, the distribution of bronchoscopy diagnosis is relatively loose and not concentrated in the upper left corner, while the distribution of CT diagnosis research is concentrated in the upper left corner.

## 4 Discussion

CT is a traditional diagnostic method that uses the correctly collimated X-ray beam, gamma ray, and ultrasonic wave to do cross-sectional scanning one by one around a certain part of the human body together with highly sensitive detectors. It has the characteristics of fast scanning time and clear images. Bronchoscopy refers to the insertion of a slender bronchoscope into the patient’s lower respiratory tract through the mouth and nasal cavity, i.e., through the glottis into the trachea and bronchus or beyond, to directly observe the lesions of the trachea and bronchus. Most lung or airway diseases can be diagnosed by bronchoscopy, but bronchoscopy is not clear for nodules in the periphery of the lung, and sometimes it is necessary to assist with thoracoscopy. At present, the pathological results of pulmonary nodule biopsy are the gold standard of diagnosis, and the comparison of CT or bronchoscope-guided pulmonary nodule biopsy is of great significance for clinical diagnosis and treatment. In this study, a meta-analysis of CT and bronchoscopic diagnosis of pulmonary nodules was performed, and relevant clinical studies in Pubmed, Embase, Cochrane Library, and Web of science databases were screened and analyzed using Revman 5.3 software. There was no significant bias in the eight included literatures. All the literatures included were comparative studies of bronchoscopy diagnosis and CT diagnosis, so the diagnostic accuracy of the two methods was comparable to a certain extent. The forest map showed that the accuracy and specificity of bronchoscopy diagnosis of pulmonary nodules were significantly better than CT diagnosis (*p* < 0.05), and the area under the ROC curve was much greater than 50%, indicating that the accuracy of these two diagnostic methods was very high, and they were suitable for clinical diagnosis of pulmonary nodules. In recent years, new methods such as CT-guided bronchoscopy or computer-assisted bronchoscopy have been applied to the diagnosis of pulmonary nodules.

As a traditional method for diagnosing pulmonary nodules, CT diagnosis has been compared with many emerging techniques. CT diagnosis is an important comparison standard, which can measure the diagnostic effect of various techniques. Herath et al. [16] compared computed tomo-guided transthoracic lung biopsy (CT-TTB) with radial-bronchial ultrasound frozen biopsy (R-EBUS, Cryo Radial) for diagnosis of peripheral pulmonary lesions. All patients in the study had nodules of >1 cm in their lungs. The results showed that Cryo-Radial had comparable diagnostic rates and epidermal growth factor receptor (EGFR) detection capabilities compared to CT-TTB, and that Cryo-Radial had the additional advantage of mediastinal staging and was a good diagnostic tool for lung cancer. These studies all show that the new technique has certain diagnostic advantages. Herath et al. [16] explored and compared the diagnostic rates of computed tomo-guided transthoracic lung biopsy (CT-TTB) with radio-bronchial ultrasound frozen biopsy (CRO-Radial) for peripheral pulmonary disease in a randomized controlled, multicenter exploratory study in three tertiary hospitals. Patients with pulmonary nodules >1 cm examined by chest CT were randomly divided into CT-TTB and CRO-Radial groups. The results showed that the diagnostic ratio was 93.8 and 85%, and the odds ratio was 0.37. The results showed an advantage in the Cryo-Radial diagnostic rate and EGFR detection capacity compared to CT-TTB, and a lower risk of emphysema. The examination and diagnosis of pulmonary nodules by CT or electromagnetically guided bronchoscopy has become a new clinical technique in recent years. The diagnosis and biopsy of pulmonary nodules by bronchoscopy are becoming more and more mature in clinical practice. Kuo et al. [17] introduced a new positioning method, which used electromagnetic navigation bronchoscope (ENB) to diagnose pulmonary nodules and compared it with CT diagnosis. From January 2016 to May 2018, 18 patients with 27 lung nodules underwent thoracoscopic pneumonectomy and 268 patients with 325 lung nodules underwent CT-guided surgery. The results of ENB-guided surgery are similar to those of CT-guided localization surgery and can effectively locate small, deep, and inaccessible lung lesions. Semaan et al. [18] used ENB to locate lung nodule patients with CT chest imaging on the same day. The study conducted navigation bronchoscopy diagnosis on 116 patients from January 2015 to June 2016, and the results showed that eight cases (6.9%) had surgery canceled due to nodule shrinkage or regression, which means the pulmonary nodules have a chance to resolve on their own. Ost et al. [19] conducted a randomized controlled study between CT fluoroscopy-guided bronchoscopy and conventional bronchoscopy in patients with suspected lung cancer. Cytologists and pathologists used blind methods for bronchial copy type. Patients with negative results received open surgical biopsy. The results showed that the sensitivity of 26 cases of CT-guided bronchoscopy and 24 cases of conventional bronchoscopy was 100 and 67%, respectively, and CT scan had higher diagnostic accuracy, especially for larger lesions. Yarmus et al. [20] conducted a prospective randomized comparative study of three bronchoscopies for pulmonary nodules, and the results showed that the diagnostic accuracy of EMN and radial intrabronchial ultrasound (EBUS) was close to 50%. The development of new technology of robotic bronchoscopy (RB) is likely to improve the evaluation of peripheral pulmonary nodules (PPNs). The study used these three bronchoscopies in a human cadaver model with PPNs < 2 cm. Sixty surgeries showed that the implanted PPNs were distributed in the lung lobes and 80% were located in non-peripheral areas. It was concluded that the use of RB significantly improved the ability to locate and puncture small PPNs in the cadaver model. Navani et al. [21] compared bronchial ultrasound-guided bronchial needle aspiration with traditional methods for the diagnosis and staging of lung cancer. In this open-label, multicenter, practical randomized controlled trial, patients from six UK centers who had received CT scans were suspected to have stage I to IIIA lung cancer. Patients were randomly assigned to either EBUS-TBNA or CDS, and the median time to treatment decision was shorter in the BUS-TBNA group (14 days; 95% CI 14–15) compared to the CDS group (29 days; the hazard ratio was 1.98 (1.39–2.82, *p* < 0.0001). This shows that for patients with suspected lung cancer, bronchial ultrasound-guided bronchial needle aspiration is used as a preliminary diagnosis method, because it can shorten the diagnosis time.

This study conducted a meta-analysis of literature comparing CT and bronchoscopy in the diagnosis of pulmonary nodules. All patients included in the study had biopsy or autopsy pathological results. This article screened literature from four databases and identified them one by one. Only eight articles were included, with a small number of articles included. In the future, there is an opportunity to search and screen as many databases as possible, especially databases from various countries. By screening literature from various countries and regions, the most objective conclusions of CT and bronchoscopy in the diagnosis of pulmonary nodules can be obtained. This is of great help to imaging in the diagnosis of pulmonary nodules.

## 5 Conclusion

A meta-analysis of several comparative studies on the diagnosis of pulmonary nodules by CT and bronchoscopy showed that the diagnostic efficiency and accuracy of the two methods were higher, but the accuracy and specificity of bronchoscopy diagnosis were significantly higher than that of CT diagnosis (*p* < 0.05), and bronchoscopy diagnosis could make up for the shortcomings of CT. The combination of the two methods may be better for diagnosing pulmonary nodules.
